# Death of Dr. G. W. Owen

**Published:** 1887-11

**Authors:** 


### EDITORIALS AND MISCELLANEOUS.

DEATH OF DR. W. G. OWEN.—Since the issue of our last journal our esteemed fellow-citizen and medical friend, Dr. W. G. Owen, of this city, departed this life. Several years ago, on account of failing health from hepatic disease, he was compelled to resign his professorship in the Southern Medical College, being in the Chair of Practice, which position he held with distinguished ability. Dr. Owen was a courtious, upright, and highly intelligent gentleman—warmly esteemed by the profession and the community at large. He leaves a beloved wife and family to mourn his loss, and his numerous patrons will greatly miss his kind and skillful attention as a practitioner, and will sadly but warmly cheerish the memory of this good and noble-hearted man.

